# Robotic nipple-sparing mastectomy complication rate compared to traditional nipple-sparing mastectomy: a systematic review and meta-analysis

**DOI:** 10.1007/s11701-021-01265-w

**Published:** 2021-06-14

**Authors:** M. D. Filipe, E. de Bock, E. L. Postma, O. W. Bastian, P. P. A. Schellekens, M. R. Vriens, A. J. Witkamp, M. C. Richir

**Affiliations:** 1 Department of Surgery, Cancer Centre, University Medical Centre Utrecht, PO Box 85500, 3508 GA Utrecht, The Netherlands; 2 Department of Surgery, St. Antonius Hospital, Nieuwegein, The Netherlands; 3 Department of Plastic Surgery, University Medical Centre Utrecht, Utrecht, The Netherlands

**Keywords:** Robot assisted nipple sparing mastectomy, Surgery, Minimally invassive

## Abstract

Breast cancer is worldwide the most common cause of cancer in women and causes the second most common cancer-related death. Nipple-sparing mastectomy (NSM) is commonly used in therapeutic and prophylactic settings. Furthermore, (preventive) mastectomies are, besides complications, also associated with psychological and cosmetic consequences. Robotic NSM (RNSM) allows for better visualization of the planes and reducing the invasiveness. The aim of this study was to compare the postoperative complication rate of RNSM to NSM. A systematic search was performed on all (R)NSM articles. The primary outcome was determining the overall postoperative complication rate of traditional NSM and RNSM. Secondary outcomes were comparing the specific postoperative complication rates: implant loss, hematoma, (flap)necrosis, infection, and seroma. Forty-nine studies containing 13,886 cases of (R)NSM were included. No statistically significant differences were found regarding postoperative complications (RNSM 3.9%, NSM 7.0%, *p* = 0.070), postoperative implant loss (RNSM 4.1%, NSM 3.2%, *p* = 0.523), hematomas (RNSM 4.3%, NSM 2.0%, *p* = 0.059), necrosis (RNSM 4.3%, NSM 7.4%, *p* = 0.230), infection (RNSM 8.3%, NSM 4.0%, *p* = 0.054) or seromas (RNSM 3.0%, NSM 2.0%, *p* = 0.421). Overall, there are no statistically significant differences in complication rates between NSM and RNSM.

**Supplementary Information:**

The online version contains supplementary material available at 10.1007/s11701-021-01265-w.

## Introduction

Breast cancer is the most common type of cancer in women and the second most common cause of death due to cancer in women worldwide [1]. There are approximately 17,000 new cases of breast cancer in the Netherlands every year. In addition, over 3,000 women of the Dutch population annually die due to breast cancer [2]. Hereditary breast cancer accounts for up to 5–10% of all breast cancers. Two high-penetrance genes (*BRCA1* and *BRCA2*) are responsible for about 16% of the familial risk of breast cancers and associated with a 60–80% lifetime risk of developing breast cancer [3, 4].

Surgical resection of the primary tumor is the treatment of choice in patients with new-onset breast cancer. Tumor stage and molecular characteristics determine the type of surgery. Most patients are treated by breast-conserving surgery followed by radiation therapy (breast-conserving therapy, BCT) or mastectomy with or without breast reconstruction [5, 6].

Currently, the ultimate prevention in women with hereditary breast cancer is bilateral prophylactic mastectomy [7]. Consequently, this means that 20–40% of these patients undergo mastectomies without signs of malignancy. Unfortunately, a part of these patients will develop complications or experience poor cosmetic results and carry a significant psychological burden [8, 9].

Nipple-sparing mastectomy (NSM) was initially reserved for the prophylactic treatment of women with a high risk of developing breast cancer [10]. However, NSM has been increasingly used therapeutically for breast cancer where the nipple-areolar complex is not involved [11–14]. One of the most important challenges of nipple-sparing mastectomy is achieving adequate exposure to performing precise dissection in areas that are remote from the skin incision [15].

Robotic nipple-sparing mastectomy (RNSM) is a relatively new technique that allows for better visualization of tissue planes and exposes tissue that is challenging to reach with traditional nipple-sparing mastectomy techniques [16–18]. Previous research has not only demonstrated the feasibility and safety of RNSM but also that RNSM has a steep learning curve [16, 19]. However, there are currently no studies comparing the complication rate of RNSM to NSM. Therefore, the aim of this study is to compare postoperative complications of patients undergoing traditional NSM to RNSM followed by immediate breast reconstruction.

## Materials and methods

This systematic review and meta-analysis were performed according to the guidelines of the requirements of the PRISMA Checklist for meta-analysis [20]. A systematic literature search was performed in the PubMed, Embase and Cochrane Library databases. The search strategy was performed on all index tests (NSM and RNSM) and their synonyms. The full electronic search strategy can be found in the supplementary data (Supplementary Material 1). After the removal of duplicates, two authors (MF, EB) independently screened articles by title and abstract. The two authors discussed discordant judgments until consensus was reached. The full articles were independently screened for eligibility based on predefined inclusion and exclusion criteria.

### Selection of studies

Full texts were retrieved for studies that evaluated (robot) nipple-sparing mastectomy, reported original data and were written in English.

1. Participants: patients undergoing therapeutic or prophylactic (R)NSM with immediate breast reconstruction (IBR).
2. Intervention: (R)NSM.
3. Outcome: postoperative complications (implant loss, hematoma, necrosis, infection or seroma).

Studies were excluded from systematic review based on the following criteria:

1. Not possible to determine whether patients had immediate reconstruction.
2. Non-robotic endoscopic NSM and/or reconstruction.
3. Intra-operative radiotherapy.
4. Case report, review and conference abstracts.

### Risk of bias

The ROBINS-I Tool was used to evaluate the quality of each eligible study [21]. The entire scale constituted seven domains for the risk of bias; confounding, selection of participants, classification of interventions, deviations from intended interventions, missing data, measurement of outcomes, and selection of the reported result. Each domain was judged for three levels of bias: low risk, intermediate/unclear risk, or high risk of bias. Full assessment criteria can be found in the supplementary data (Supplementary Material 2).

### Statistical analysis

Pairwise meta-analysis was performed to compare complication rates with 95% confidence intervals (CI) of RNSM to NSM in studies. Pooled postoperative complication rates were determined using random effects models. *p* values under 0.05 were considered statistically significant.

All calculations were performed using RStudio 1.2.5001 (with R version: ×64 3.6.3). Additionally, statistical packages *meta*, *mada*, *metafor*, *gemtc*, *mvmeta* and were used for all computations of the meta-analyses. Visualization of plots was done using the *ggplot2* package.

## Results

**Fig. 1:**
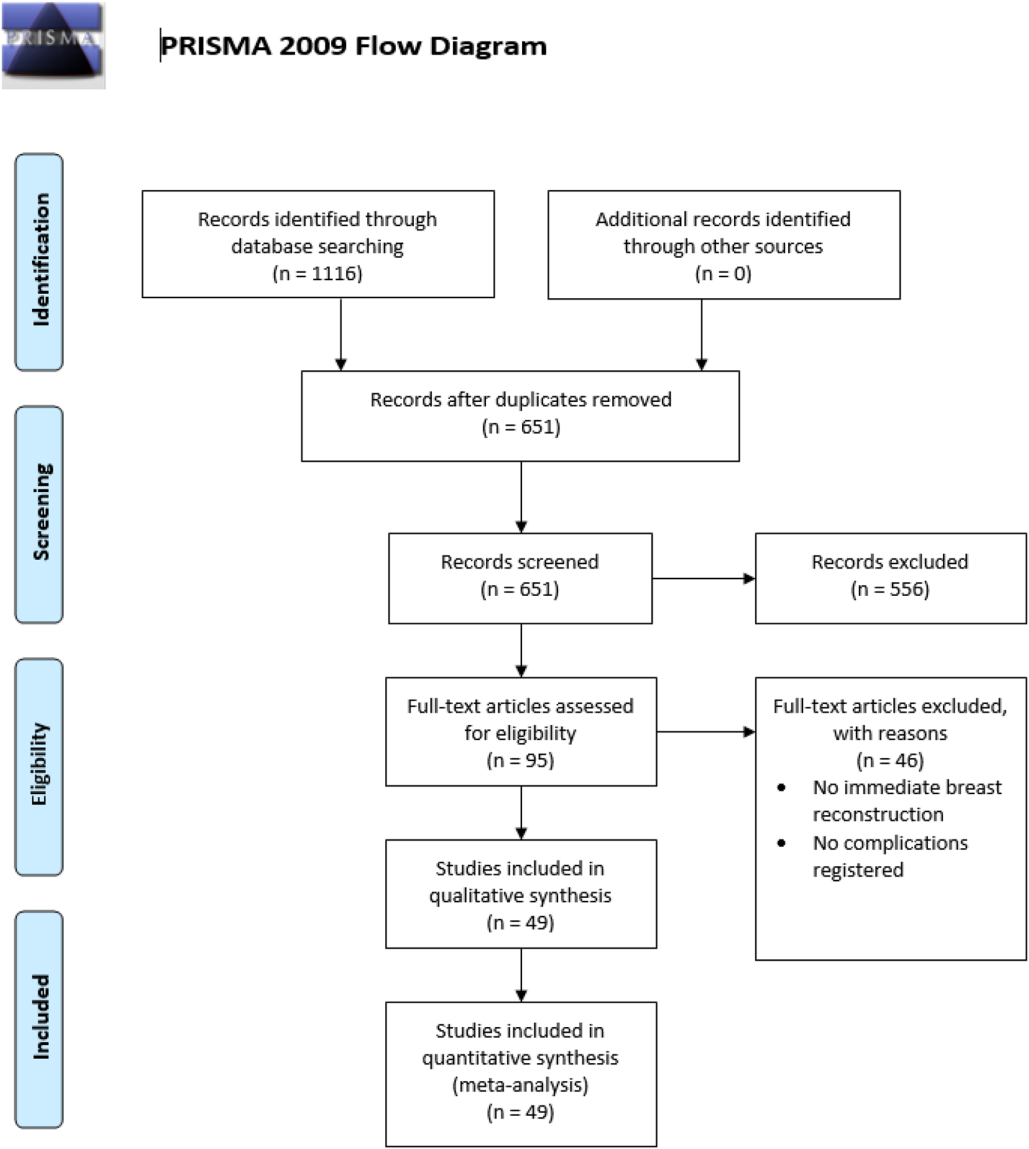
Flow chart showing literature search and study selection with 49 relevant studies ultimately enrolled in this meta-analysis on the complication rate of (robotic) nipple-sparing mastectomy

One thousand one hundred and sixteen citations were identified by the search and, after removing duplicates, 95 potentially eligible articles were retrieved in full text (Fig. 1). Overall, 13,886 (R)NSM were performed in 49 studies with an average of 294.6 participants per study [supplementary Table 1].

**Fig. 2:**
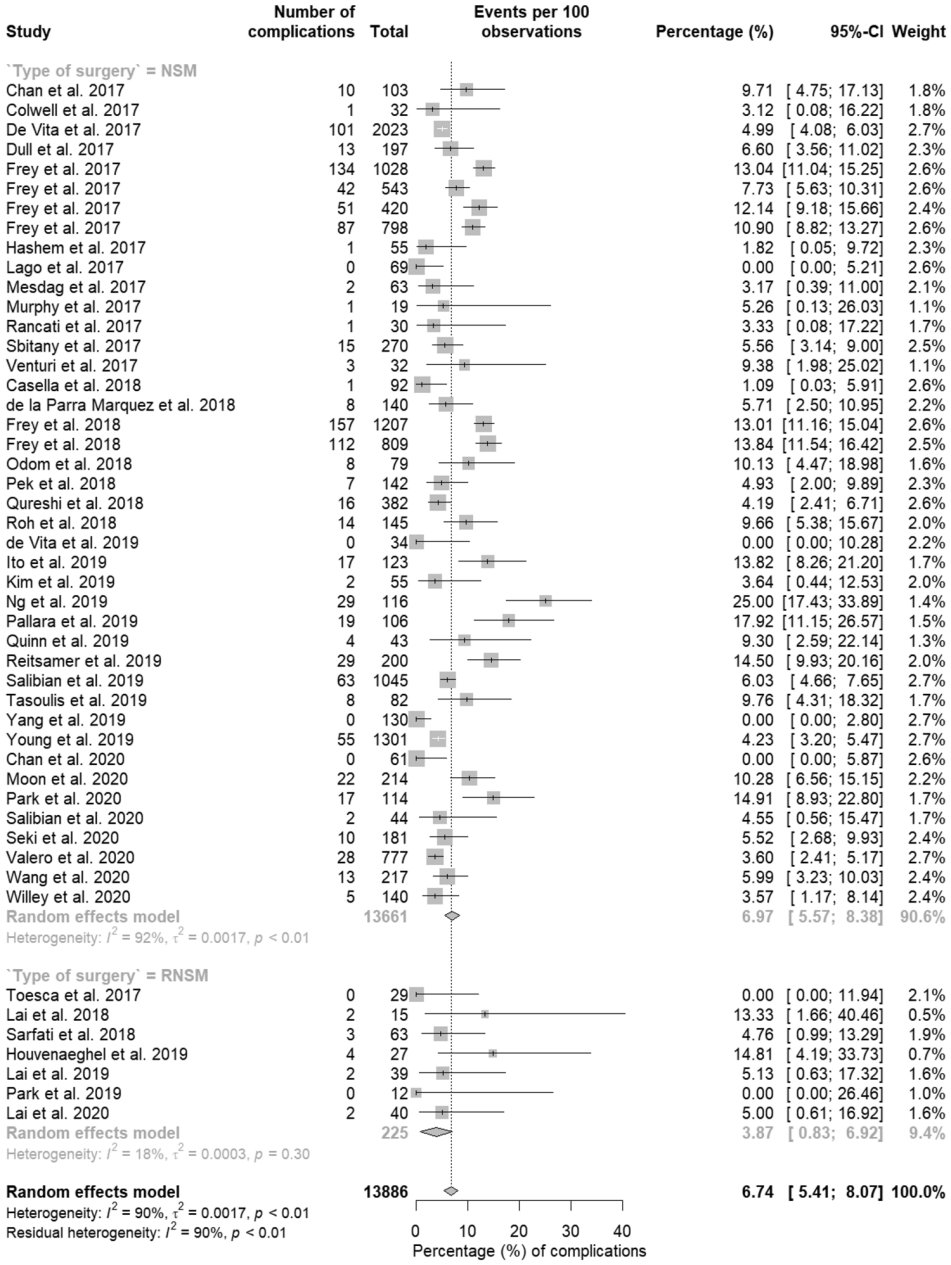
Complication rates. *(R)NSM* (robotic) nipple-sparing mastectomy, *CI* confidence interval

Supplementary Table 1 shows the studies included in the analysis and their characteristics. Seven studies described postoperative complications of RNSM and 42 studies described the postoperative complications of NSM. In total, 13 out of 225 mastectomies (3.9%) developed postoperative complications in RNSM while 1,056 out of 13,661 NSM (7.0%) developed postoperative complications. This difference was not statistically significant (*p* = 0.070) (Fig. 2).

### Different postoperative complications

Detailed meta-analysis of the different complications of each study can be found in Supplementary Figs. 1–5. Post-mastectomy implant removal occurred in 4.1% of RNSM and in 3.2% in NSM. This difference was not statistically significant (*p* = 0.523). Furthermore, post-mastectomy hematoma occurred more often in RNSM (4.3%) than in NSM (2.0%) but this difference was not statistically significant (*p* = 0.059). Moreover, post-mastectomy necrosis and seroma occurred in respectively 4.3% and 3.0% in RNSM and 7.4% and 2.0% in NSM. These differences were not statistically significant. Finally, postoperative mastectomy infection occurred more often in RNSM (8.3%) than in NSM (4.0%) but this was not statistically significant (*p* = 0.054) (Table 1).

**Table 1:** Pooled complication rates of (R)NSM

Parameter, % (95% CI)	RNSM (%)	NSM (%)	*p* value
Total complications	3.9 (0.8–6.9)	7.0 (5.6–8.4)	0.070
Implant loss	4.1 (1.9–8.7)	3.2 (2.4–4.2)	0.523
Hematoma	4.3 (2.0–9.1)	2.0 (1.7–2.4)	0.059
Necrosis	4.3 (1.8–10.0)	7.4 (5.8–9.3)	0.230
Infection	8.3 (4.2–15.8)	4.0 (3.0–5.3)	0.054
Seroma	3.0 (1.3–7.1)	2.0 (1.3–3.1)	0.421

*(R)NSM* (robotic) nipple-sparing mastectomy, *CI* confidence interval

### Risk of bias

**Fig. 3:**
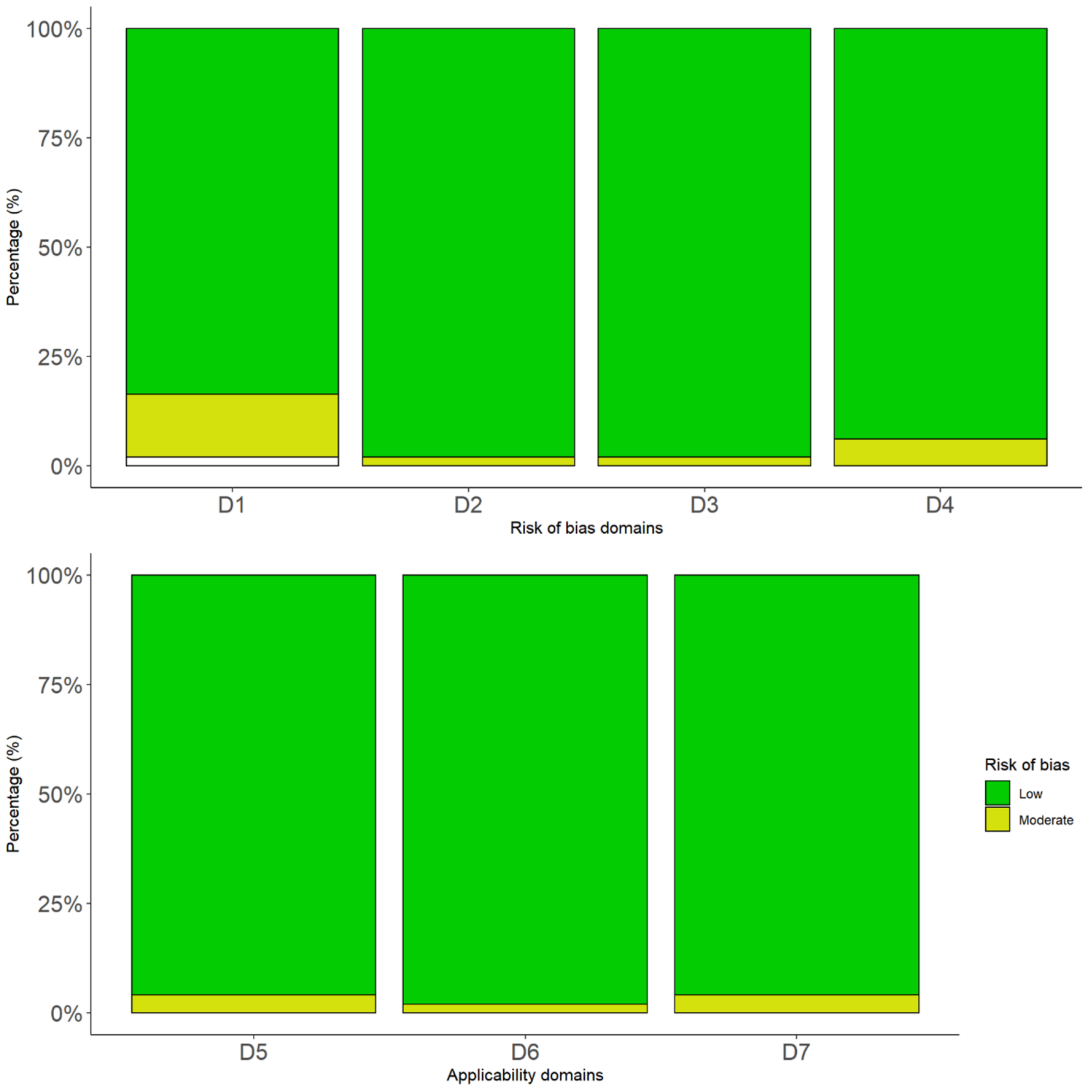
Summary of the risks of bias and applicability domains. D1 = Bias due to confounding D2 = Bias in selection of participants into the study; D3 = Bias in the classification of interventions; D4 = Bias due to deviations from intended interventions; D5 = Bias due to missing data; D6 = Bias in measurements of outcomes; D7 = Bias in the selection of the reported result

The result of the ROBINS-I Tool revealed that all the included studies were of sufficient quality. This was for risks of bias domains and applicability domains (Fig. 3). Risk assessment of every study can be found in Supplementary Material 4.

## Discussion

This meta-analysis, including 49 studies, is the first study to compare the complication rate of RNSM to NSM in patients undergoing prophylactic or therapeutic mastectomy. The current study shows that patients undergoing RNSM do not have an increased risk of developing postoperative complications when compared to NSM.

Approximately 40% of patients with invasive breast cancer and 30% of patients with ductal carcinoma in situ (DCIS) undergo mastectomy [22]. Additionally, there are many BRCA1 or BRCA2 gene mutation carriers that undergo prophylactic mastectomies. As mastectomies are accompanied by complications along with serious cosmetic and psychological consequences [8, 9]. For these patients, it is essential to keep the surgical intervention as minimally invasive as possible, while preserving the (oncological) safety. RNSM has shown to be feasible and safe, both as a therapeutic and a prophylactic treatment [19, 23]. To obtain information to provide a reliable insight into the postoperative complications between NSM and RNSM procedures, a literature search was performed. The overall complication rate was 3.9% after RNSM and 7.0% after NSM. This difference was not statistically different. Considering individual complications, patients did not appear to have an increased risk of postoperative implant loss or developing hematomas, infections, seromas or necrosis due to RNSM.

While there are no differences in complications between traditional NSM and RNSM, RNSM does offer certain advantages. Robotic surgery in general provides smaller incisions compared to open surgery [24]. Furthermore, RNSM allows for better visualization of the planes and exposes tissue that is challenging to reach with traditional nipple-sparing mastectomy techniques [16–18]. Another advantage of the scopic nature of the RNSM could be that this technique allows enhanced imaging techniques that could detect (pre)cancerous breast cancer lesions. One of these techniques is narrow-band imaging (NBI). NBI is a well-established technique used during colonoscopy, cystoscopy, and bronchoscopy to detect (pre)cancerous lesions of epithelial origin [25–27]. Therefore, it is reasonable to assume that NBI might be useful since (pre)malignancy is known to show different patterns of vascularization compared to healthy breast tissue [28, 29]. Furthermore, (pre)malignant epithelial lesions show an aberrant pattern under fluorescent light by which they become detectable. This technique is already used extensively to help identify pathologies within the airways, larynx, and colon [30–32]. Moreover, studies have also shown promising results of (auto) fluorescence for the detection of (pre)cancerous lesions of the breast [33, 34]. Consequently, enhanced imaging techniques, such as NBI and (auto) fluorescence, combined with robotic surgery could improve margin determination in patients undergoing breast cancer surgery. Furthermore, another advantage of robotic surgery is that it provides better ergonomics for the surgeon when compared to traditional surgery [35]. A possible downside of robotic breast surgery, as with many other types of robotic surgery, is that the preparations (docking and positioning of the patient) and the procedure itself takes longer [16]. The longer operation time results, along with the higher material costs, in an overall more expensive procedure.

A recent systematic review also concluded that RNSM is safe to use with acceptable short-term outcomes [36]. However, the current study quantifies (by means of a meta-analysis) the risk of postoperative complications in patients undergoing RNSM and compares said risk to traditional mastectomy.

This study has some limitations. The number of patients in the studies describing RNSM are relatively low compared to traditional NSM. This can be attributed to the fact that RNSM is a relatively new technique. Furthermore, the current study did not describe which percentage of the mastectomies were prophylactic or therapeutic. This might have an effect on the risk of postoperative complications since neo-adjuvant therapy increases the risk of postoperative complications [37]. Although RNSM has great potential, additional prospective research is warranted to further determine oncological safety, long-term postoperative complications and patient-reported outcomes in patients undergoing RNSM. Currently, in most countries RNSM is off-label. Therefore, the previously proposed additional research and the results of the current study could help to make RNSM a recommended viable option for women requiring mastectomy.

In conclusion, this study shows that there is no significant difference in the postoperative complication rate of RNSM compared to traditional NSM. Therefore, RNSM can be used safely in patients that require a prophylactic or therapeutic mastectomy.

## Supplementary Information

Below is the link to the electronic supplementary material.

Supplementary file1 (DOCX 5200 KB)
