# Supplementary material for: Robotic nipple-sparing mastectomy complication rate compared to traditional nipple-sparing mastectomy: a systematic review and meta-analysis
Source: J Robot Surg. 2021 Jun 14;16(2):265–72. doi: 10.1007/s11701-021-01265-w (PMC8960562; doi:10.1007/s11701-021-01265-w)
Supplement: Supplementary file 1 — Supplementary file1 (DOCX 5200 KB) [file 11701_2021_1265_MOESM1_ESM.docx]

Robotic nipple sparing mastectomy complication rate compared to traditional nipple sparing mastectomy: a systematic review and meta-analysis.

M.D. Filipe^1^ MD m.d.filipe@umcutrecht.nl

E. de Bock^1^ BSc

E.L. Postma^2^ MD PhD

O.W. Bastian^1^ MD PhD

P.P.A. Schellekens^3^ MD PhD

M.R. Vriens^1^ MD PhD

A.J. Witkamp^1^ MD PhD

M.C. Richir^1^ MD PhD m.c.richir@umcutrecht.nl

^1^ Department of Surgery, Cancer Centre, University Medical Centre Utrecht, Utrecht, The Netherlands

^2^ Department of Surgery, St. Antonius Hospital, Nieuwegein, the Netherlands

^3^Departments of Plastic Surgery, University Medical Centre Utrecht, Utrecht, the Netherlands

Corresponding author

M.D. Filipe

Address: PO Box 85500, 3508 GA, Utrecht, The Netherlands

E-mail: [m.d.filipe@umcutrecht.nl](mailto:m.d.filipe@umcutrecht.nl)

# Supplementary Material 1: Search strategy

Search 28/2/2020

PUBMED:

**(("Mastectomy"[Mesh] OR Mastectom*[Title/Abstract]) AND ("Nipples"[Mesh] OR nipple[Title/Abstract] OR nipples[Title/Abstract]) AND sparing[Title/Abstract])**

EMBASE:

('mastectomy'/exp OR Mastectom*:ti,ab,kw) AND ('nipple'/exp OR nipple:ti,ab,kw OR nipples:ti,ab,kw) AND (sparing:ti,ab,kw)

# Supplementary Material 2: ROBINS-I risk of bias tool

Responses underlined in green are potential markers for low risk of bias, and responses in red are potential markers for a risk of bias. Where questions relate only to sign posts to other questions, no formatting is used.

|  | **Signalling questions** | **Description** | **Response options** |
| --- | --- | --- | --- |
| **Bias due to confounding** | | | |
|  | 1.1 Is there potential for confounding of the effect of intervention in this study?  **If N/PN to 1.1:** the study can be considered to be at low risk of bias due to confounding and no further signalling questions need be considered |  | Y / PY / PN / N |
|  | **If Y/PY to 1.1**: determine whether there is a need to assess time-varying confounding: |  |  |
|  | 1.2. Was the analysis based on splitting participants’ follow up time according to intervention received?  **If N/PN**, answer questions relating to baseline confounding (1.4 to 1.6)  **If Y/PY**, go to question 1.3. |  | NA / Y / PY / PN / N / NI |
|  | 1.3. Were intervention discontinuations or switches likely to be related to factors that are prognostic for the outcome?  **If N/PN**, answer questions relating to baseline confounding (1.4 to 1.6)  **If Y/PY**, answer questions relating to both baseline and time-varying confounding (1.7 and 1.8) |  | NA / Y / PY / PN / N / NI |

|  | **Questions relating to baseline confounding only** | | |
| --- | --- | --- | --- |
|  | 1.4. Did the authors use an appropriate analysis method that controlled for all the important confounding domains? |  | NA / Y / PY / PN / N / NI |
|  | 1.5. **If Y/PY to 1.4**: Were confounding domains that were controlled for measured validly and reliably by the variables available in this study? |  | NA / Y / PY / PN / N / NI |
|  | 1.6. Did the authors control for any post-intervention variables that could have been affected by the intervention? |  | NA / Y / PY / PN / N / NI |
|  | **Questions relating to baseline and time-varying confounding** | |  |
|  | 1.7. Did the authors use an appropriate analysis method that controlled for all the important confounding domains and for time-varying confounding? |  | NA / Y / PY / PN / N / NI |
|  | 1.8. **If Y/PY to 1.7**: Were confounding domains that were controlled for measured validly and reliably by the variables available in this study? |  | NA / Y / PY / PN / N / NI |
|  | **Risk of bias judgement** |  | Low / Moderate / Serious / Critical / NI |
|  | Optional: What is the predicted direction of bias due to confounding? |  | Favours experimental / Favours comparator / Unpredictable |

| **Bias in selection of participants into the study** | | | |
| --- | --- | --- | --- |
|  | 2.1. Was selection of participants into the study (or into the analysis) based on participant characteristics observed after the start of intervention?  **If N/PN to 2.1:** go to 2.4 |  | Y / PY / PN / N / NI |
|  | 2.2. **If Y/PY to 2.1**: Were the post-intervention variables that influenced selection likely to be associated with intervention?  2.3 **If Y/PY to 2.2**: Were the post-intervention variables that influenced selection likely to be influenced by the outcome or a cause of the outcome? |  | NA / Y / PY / PN / N / NI  NA / Y / PY / PN / N / NI |
|  | 2.4. Do start of follow-up and start of intervention coincide for most participants? |  | Y / PY / PN / N / NI |
|  | 2.5. **If Y/PY to 2.2 and 2.3, or N/PN to 2.4**: Were adjustment techniques used that are likely to correct for the presence of selection biases? |  | NA / Y / PY / PN / N / NI |
|  | **Risk of bias judgement** |  | Low / Moderate / Serious / Critical / NI |
|  | Optional: What is the predicted direction of bias due to selection of participants into the study? |  | Favours experimental / Favours comparator / Towards null /Away from null / Unpredictable |

| **Bias in classification of interventions** | | | |
| --- | --- | --- | --- |
|  | 3.1 Were intervention groups clearly defined? |  | Y / PY / PN / N / NI |
|  | 3.2 Was the information used to define intervention groups recorded at the start of the intervention? |  | Y / PY / PN / N / NI |
|  | 3.3 Could classification of intervention status have been affected by knowledge of the outcome or risk of the outcome? |  | Y / PY / PN / N / NI |
|  | **Risk of bias judgement** |  | Low / Moderate / Serious / Critical / NI |
|  | Optional: What is the predicted direction of bias due to classification of interventions? |  | Favours experimental / Favours comparator / Towards null /Away from null / Unpredictable |

| **Bias due to deviations from intended interventions** | | | |
| --- | --- | --- | --- |
|  | **If your aim for this study is to assess the effect of assignment to intervention, answer questions 4.1 and 4.2** | |  |
|  | 4.1. Were there deviations from the intended intervention beyond what would be expected in usual practice? |  | Y / PY / PN / N / NI |
|  | 4.2. **If Y/PY to 4.1**: Were these deviations from intended intervention unbalanced between groups *and* likely to have affected the outcome? |  | NA / Y / PY / PN / N / NI |
|  | **If your aim for this study is to assess the effect of starting and adhering to intervention, answer questions 4.3 to 4.6** | |  |
|  | 4.3. Were important co-interventions balanced across intervention groups? |  | Y / PY / PN / N / NI |
|  | 4.4. Was the intervention implemented successfully for most participants? |  | Y / PY / PN / N / NI |
|  | 4.5. Did study participants adhere to the assigned intervention regimen? |  | Y / PY / PN / N / NI |
|  | 4.6. **If N/PN to 4.3, 4.4 or 4.5**: Was an appropriate analysis used to estimate the effect of starting and adhering to the intervention? |  | NA / Y / PY / PN / N / NI |
|  | **Risk of bias judgement** |  | Low / Moderate / Serious / Critical / NI |
|  | Optional: What is the predicted direction of bias due to deviations from the intended interventions? |  | Favours experimental / Favours comparator / Towards null /Away from null / Unpredictable |

| **Bias due to missing data** | | | |
| --- | --- | --- | --- |
|  | 5.1 Were outcome data available for all, or nearly all, participants? |  | Y / PY / PN / N / NI |
|  | 5.2 Were participants excluded due to missing data on intervention status? |  | Y / PY / PN / N / NI |
|  | 5.3 Were participants excluded due to missing data on other variables needed for the analysis? |  | Y / PY / PN / N / NI |
|  | 5.4 **If PN/N to 5.1, or Y/PY to 5.2 or 5.3**: Are the proportion of participants and reasons for missing data similar across interventions? |  | NA / Y / PY / PN / N / NI |
|  | 5.5 **If PN/N to 5.1, or Y/PY to 5.2 or 5.3**: Is there evidence that results were robust to the presence of missing data? |  | NA / Y / PY / PN / N / NI |
|  | **Risk of bias judgement** |  | Low / Moderate / Serious / Critical / NI |
|  | Optional: What is the predicted direction of bias due to missing data? |  | Favours experimental / Favours comparator / Towards null /Away from null / Unpredictable |

| **Bias in measurement of outcomes** | | | |
| --- | --- | --- | --- |
|  | 6.1 Could the outcome measure have been influenced by knowledge of the intervention received? |  | Y / PY / PN / N / NI |
|  | 6.2 Were outcome assessors aware of the intervention received by study participants? |  | Y / PY / PN / N / NI |
|  | 6.3 Were the methods of outcome assessment comparable across intervention groups? |  | Y / PY / PN / N / NI |
|  | 6.4 Were any systematic errors in measurement of the outcome related to intervention received? |  | Y / PY / PN / N / NI |
|  | **Risk of bias judgement** |  | Low / Moderate / Serious / Critical / NI |
|  | Optional: What is the predicted direction of bias due to measurement of outcomes? |  | Favours experimental / Favours comparator / Towards null /Away from null / Unpredictable |

| **Bias in selection of the reported result** | | | |
| --- | --- | --- | --- |
|  | Is the reported effect estimate likely to be selected, on the basis of the results, from... |  |  |
|  | 7.1. ... multiple outcome *measurements* within the outcome domain? |  | Y / PY / PN / N / NI |
|  | 7.2 ... multiple *analyses* of the intervention-outcome relationship? |  | Y / PY / PN / N / NI |
|  | 7.3 ... different *subgroups*? |  | Y / PY / PN / N / NI |
|  | **Risk of bias judgement** |  | Low / Moderate / Serious / Critical / NI |
|  | Optional: What is the predicted direction of bias due to selection of the reported result? |  | Favours experimental / Favours comparator / Towards null /Away from null / Unpredictable |

| **Overall bias** | | | |
| --- | --- | --- | --- |
|  | **Risk of bias judgement** |  | Low / Moderate / Serious / Critical / NI |
|  | Optional: What is the overall predicted direction of bias for this outcome? |  | Favours experimental / Favours comparator / Towards null /Away from null / Unpredictable |

# Supplementary Material 3: studies included in the analysis

| Supplementary Table 1. Baseline characteristics of studies on nipple sparing mastectomy | | | | | | | | | |
| --- | --- | --- | --- | --- | --- | --- | --- | --- | --- |
| Author | year | country | type | NSM | C1 | C2 | C3 | C4 | C5 |
| Frey et al. (1) | 2017 | USA | NSM | 1028 | A | B | C | D | E |
| Rancati et al. (2) | 2017 | Argentina | NSM | 30 | B | C | D |  |  |
| Frey et al. (3) | 2017 | USA | NSM | 543 | A | B | C | D | E |
| Frey et al. (4) | 2017 | USA | NSM | 420 | D |  |  |  |  |
| Mesdag et al. (5) | 2017 | France | NSM | 63 | A | C | D | E |  |
| Dull et al. (6) | 2017 | USA | NSM | 197 | A | B | C | D | E |
| Lago et al. (7) | 2017 | Spain | NSM | 69 | D |  |  |  |  |
| Murphy et al. (8) | 2017 | USA | NSM | 19 | B | D |  |  |  |
| Chan et al. (9) | 2017 | Hong Kong | NSM | 103 | C | E |  |  |  |
| Colwell et al. (10) | 2017 | USA | NSM | 32 | A | B | C | D | E |
| De Vita et al. (11) | 2017 | Italy | NSM | 2023 | A | B | C | D | E |
| Sbitany et al. (12) | 2017 | USA | NSM | 270 | A | B | C | D | E |
| Toesca et al. (13) | 2017 | Italy | RNSM | **29** | A | B | C | D | E |
| Venturi et al. (14) | 2017 | USA | NSM | 32 | D | E |  |  |  |
| Frey et al. (15) | 2017 | USA | NSM | 798 | A | B | C | D | E |
| Hashem et al. (16) | 2017 | Egypt | NSM | 55 | A |  |  |  |  |
| Casella et al. (17) | 2018 | Italy | NSM | 92 | A | B | C | D | E |
| de la Parra Marquez et al. (18) | 2018 | Mexico | NSM | 140 | B | C | D | E |  |
| Frey et al. (19) | 2018 | USA | NSM | 1207 | A | B | C | D | E |
| Frey et al. (20) | 2018 | USA | NSM | 809 | A | B | C | D | E |
| Lai et al. (21) | 2018 | Taiwan | RNSM | **15** | A | B | C | D | E |
| Odom et al. (22) | 2018 | USA | NSM | 79 | A | B | C | D | E |
| Pek et al. (23) | 2018 | Singapore | NSM | 142 | A | C |  |  |  |
| Qureshi et al. (24) | 2018 | USA | NSM | 382 | A | B | C | E |  |
| Roh et al. (25) | 2018 | South Korea | NSM | 145 | A | B | C | D | E |
| Sarfati et al. (26) | 2018 | France | RNSM | **63** | A | B | C | E |  |
| de Vita et al. (27) | 2019 | Italy | NSM | 34 | A | B | C | D |  |
| Houvenaeghel et al. (28) | 2019 | France | RNSM | **27** | A | C | E |  |  |
| Ito et al. (29) | 2019 | Japan | NSM | 123 | D |  |  |  |  |
| Kim et al. (30) | 2019 | South Korea | NSM | 55 | A | B | C | E |  |
| Lai et al.(31) | 2019 | Taiwan | RNSM | **39** | A | B | D | E |  |
| Ng et al. (32) | 2019 | Canada | NSM | 116 | A | B | D | E |  |
| Pallara et al. (33) | 2019 | Italy | NSM | 106 | A | B | C | D | E |
| Park et al. (34) | 2019 | South Korea | RNSM | **12** | A | B | C | E |  |
| Quinn et al. (35) | 2019 | Ireland | NSM | 43 | A | C | E |  |  |
| Reitsamer et al. (36) | 2019 | Austria | NSM | 200 | A | B | E |  |  |
| Salibian et al. (37) | 2019 | USA | NSM | 1045 | A | B | C | D | E |
| Tasoulis et al. (38) | 2019 | United Kingdom | NSM | 82 | C | D | E |  |  |
| Yang et al. (39) | 2019 | China | NSM | 130 | C | D |  |  |  |
| Young et al. (40) | 2019 | USA | NSM | 1301 | A | B | C | D |  |
| Chan et al. (41) | 2020 | Hong Kong | NSM | 61 | E |  |  |  |  |
| Lai et al. (42) | 2020 | Taiwan | RNSM | **40** | A | B | D | E |  |
| Moon et al. (43) | 2020 | South Korea | NSM | 214 | A | B | D |  |  |
| Park et al. (44) | 2020 | USA | NSM | 114 | A | B | C | D | E |
| Salibian et al. (45) | 2020 | USA | NSM | 44 | A | C | D |  |  |
| Seki et al. (46) | 2020 | Japan | NSM | 181 | A | C | D | E |  |
| Valero et al. (47) | 2020 | USA | NSM | 777 | A | C | E |  |  |
| Wang et al. (48) | 2020 | USA | NSM | 217 | B | C | D |  |  |
| Willey et al. (49) | 2020 | USA | NSM | 140 | A | B | C | E |  |
| C# = Complication, A = hematoma, B = seroma, C = infection, D= flap necrosis, E = implant removal, NSM = nipple sparing mastectomy, RNSM = robot assisted nipple sparing mastectomy, USA = United States of America. | | | | | | | | | |

# Supplementary Material 4: Risk of bias assessment

| Supplementary Table2. Risk of bias for different studies. | | | | | | | |
| --- | --- | --- | --- | --- | --- | --- | --- |
| author | D1 | D2 | D3 | D4 | D5 | D6 | D7 |
| Houvenaeghel | Moderate | Low | Low | Moderate | Low | Low | Low |
| de Vita | Low | Low | Low | Low | Low | Low | Low |
| Kim | Low | Low | Low | Low | Moderate | Low | Low |
| Roh | Low | Low | Low | Low | Low | Low | Low |
| Pallara | Moderate | Low | Low | Low | Low | Low | Low |
| Pek | Low | Low | Low | Low | Low | Low | Low |
| Quinn | Low | Low | Low | Low | Low | Low | Low |
| de la Parra Marquez | Low | Low | Low | Low | Low | Low | Low |
| Frey | Low | Low | Low | Low | Low | Low | Low |
| Valero | Low | Low | Low | Low | Low | Low | Low |
| Wang | Low | Low | Low | Low | Low | Low | Low |
| Salibian | Low | Low | Low | Low | Low | Low | Moderate |
| Ng | Low | Low | Low | Low | Low | Low | Low |
| Casella | Low | Low | Low | Low | Low | Low | Low |
| Odom | Moderate | Low | Low | Low | Low | Moderate | Low |
| Young | Moderate | Low | Low | Moderate | Low | Low | Low |
| Willey | Low | Low | Low | Low | Low | Low | Low |
| Moon | Moderate | Low | Moderate | Low | Low | Low | Low |
| Reitsamer | Low | Low | Low | Low | Low | Low | Low |
| Salibian | Moderate | Moderate | Low | Moderate | Low | Low | Moderate |
| Ito | Low | Low | Low | Low | Low | Low | Low |
| Lai | Low | Low | Low | Low | Low | Low | Low |
| Park | Low | Low | Low | Low | Low | Low | Low |
| Sarfati | Low | Low | Low | Low | Low | Low | Low |
| Lai | Low | Low | Low | Low | Low | Low | Low |
| Qureshi | Low | Low | Low | Low | Moderate | Low | Low |
| Frey | Low | Low | Low | Low | Low | Low | Low |
| Lai | Low | Low | Low | Low | Low | Low | Low |
| Yang | Low | Low | Low | Low | Low | Low | Low |
| Tasoulis | Low | Low | Low | Low | Low | Low | Low |
| Chan | Low | Low | Low | Low | Low | Low | Low |
| Park | Low | Low | Low | Low | Low | Low | Low |
| Seki | Low | Low | Low | Low | Low | Low | Low |
| Frey | Low | Low | Low | Low | Low | Low | Low |
| Rancati | Low | Low | Low | Low | Low | Low | Low |
| Frey | Low | Low | Low | Low | Low | Low | Low |
| Frey | Low | Low | Low | Low | Low | Low | Low |
| Mesdag | Low | Low | Low | Low | Low | Low | Low |
| Dull | Low | Low | Low | Low | Low | Low | Low |
| Lago | Low | Low | Low | Low | Low | Low | Low |
| Murphy | Low | Low | Low | Low | Low | Low | Low |
| Chan | Low | Low | Low | Low | Low | Low | Low |
| Colwell | Low | Low | Low | Low | Low | Low | Low |
| De Vita | Low | Low | Low | Low | Low | Low | Low |
| Sbitany | Low | Low | Low | Low | Low | Low | Low |
| Toesca | Low | Low | Low | Low | Low | Low | Low |
| Venturi | Low | Low | Low | Low | Low | Low | Low |
| Frey | Moderate | Low | Low | Low | Low | Low | Low |
| Hashem | Low | Low | Low | Low | Low | Low | Low |
| D1= Bias due to confounding D2 = Bias in selection of participants into the study; D3 = Bias in classification of interventions; D4 = Bias due to deviations from intended interventions; D5 = Bias due to missing data; D6 = Bias in measurements of outcomes; D7 = Bias in selection of the reported result. | | | | | | | |

# Supplementary Material 5: supplementary figures

| 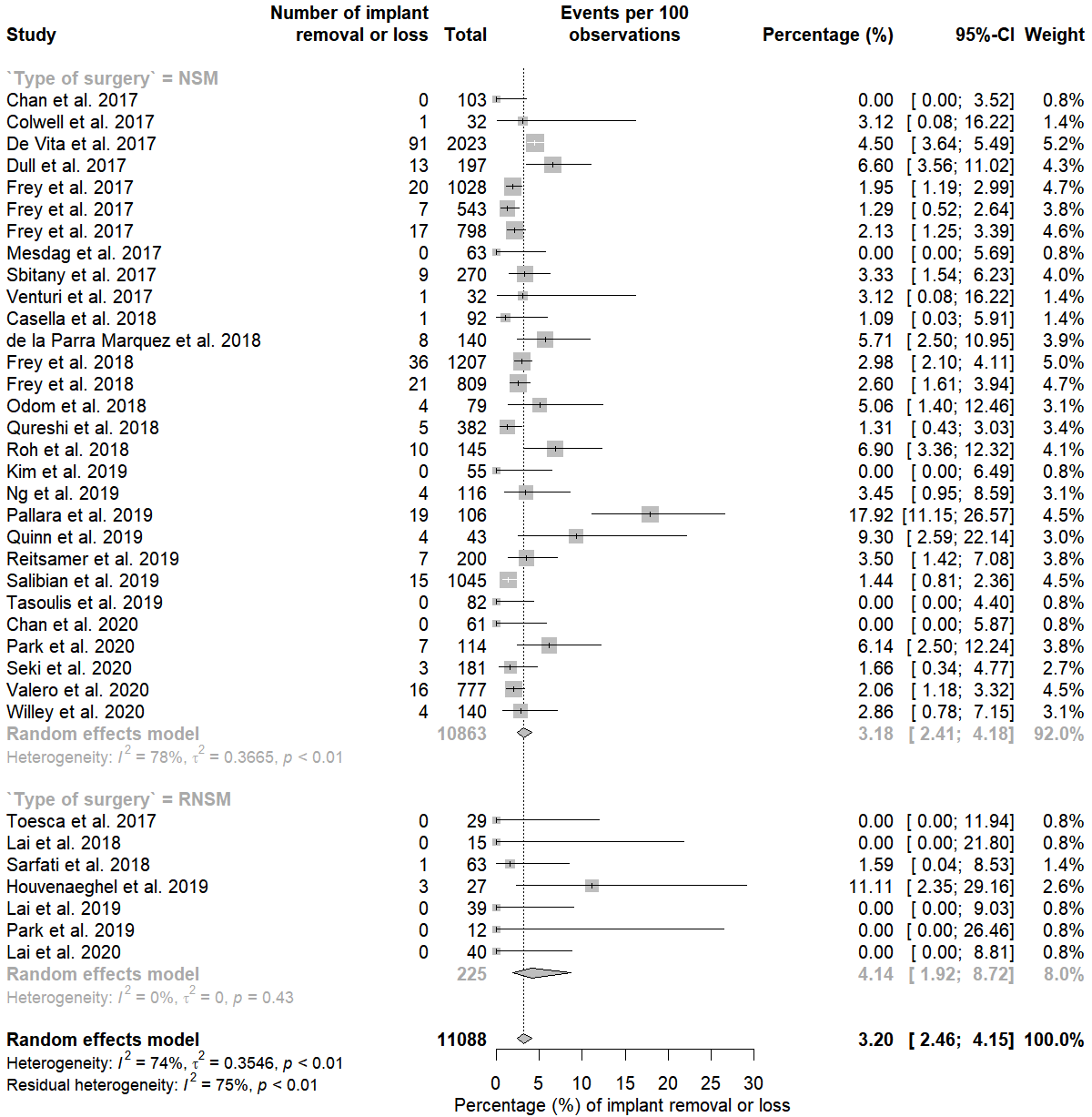 |
| --- |
| Supplementary Figure 1. Postoperative Implant loss. P = 0.523, (R)NSM = (Robotic) nipple sparing mastectomy, CI = confidence interval. |
| 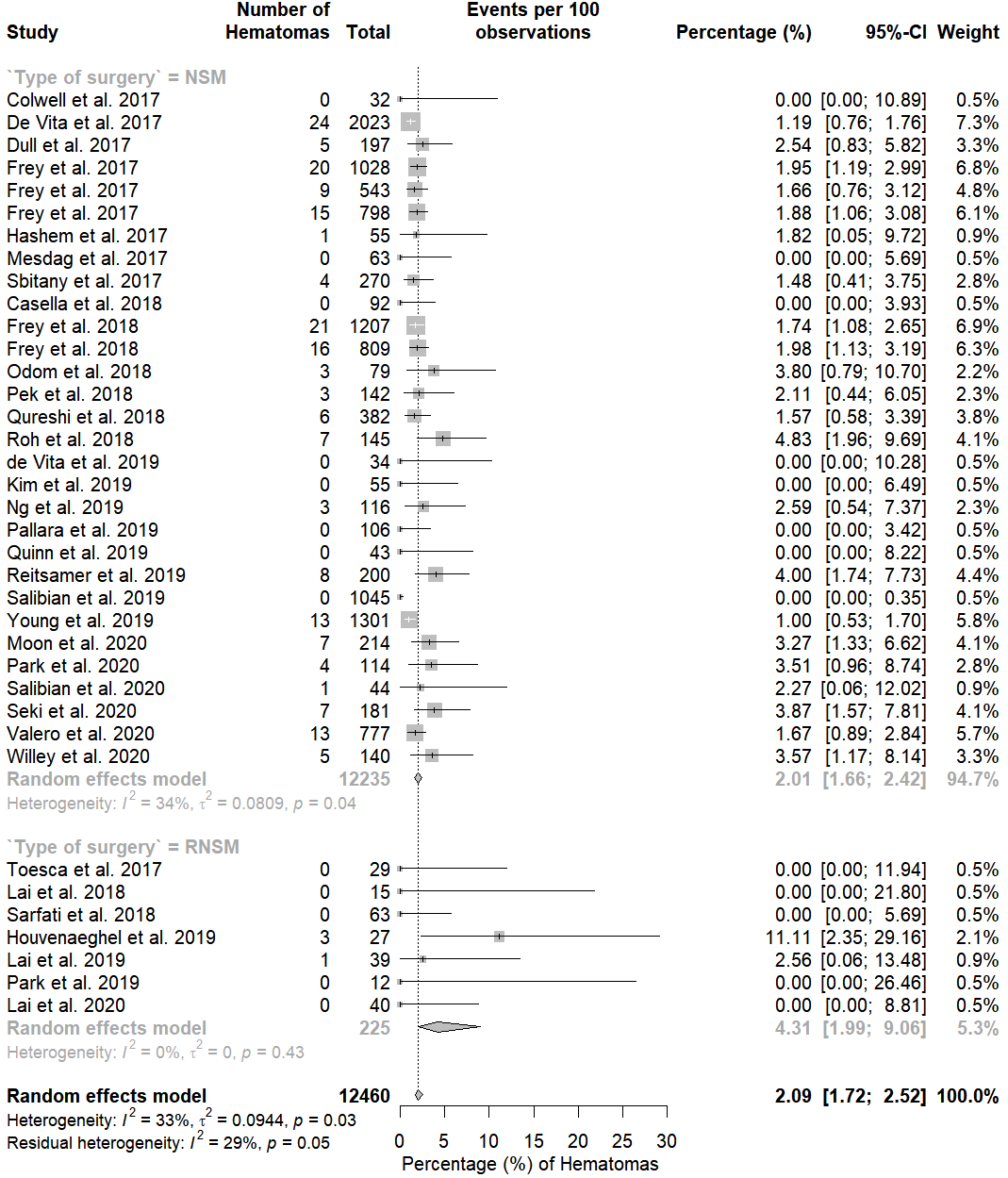 |
| Supplementary Figure 2. Hematomas. P = 0.059, (R)NSM = (Robotic) nipple sparing mastectomy, CI = confidence interval. |

| **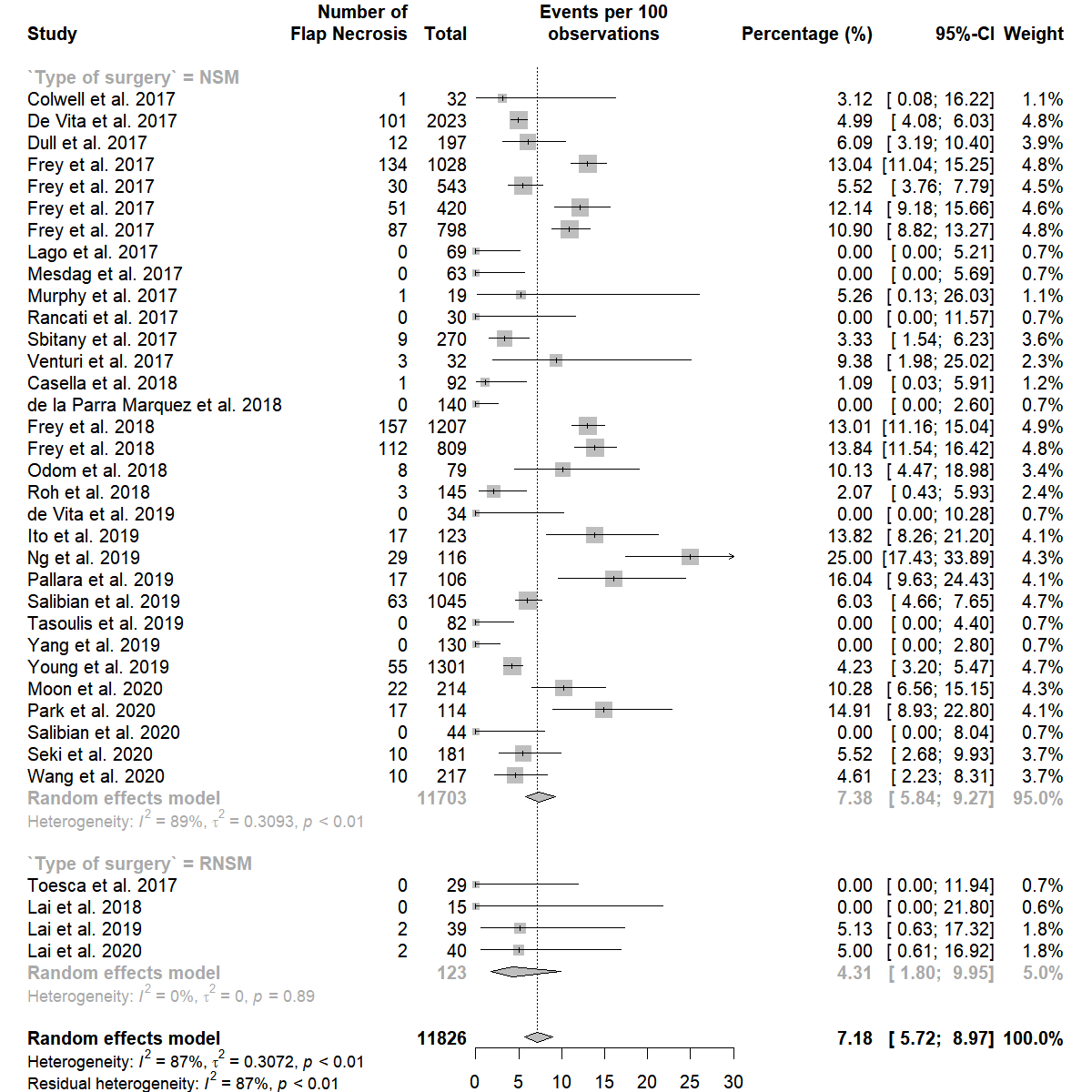** |
| --- |
| Supplementary Figure 3. Flap necrosis. P = 0.230, (R)NSM = (Robotic) nipple sparing mastectomy, CI = confidence interval. |

| 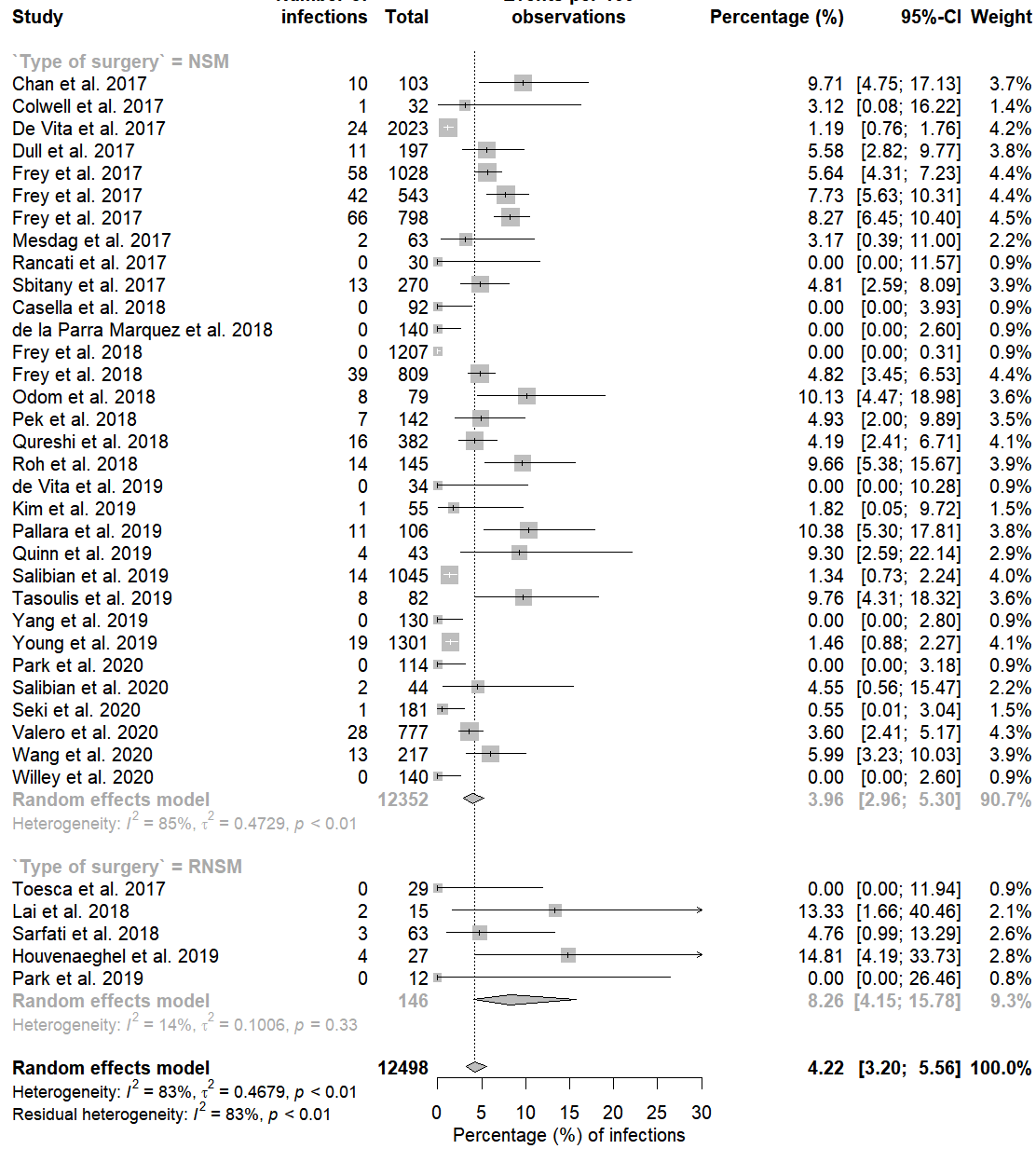 |
| --- |
| Supplementary Figure 4. Infections. P = 0.054, (R)NSM = (Robotic) nipple sparing mastectomy, CI = confidence interval. |

| 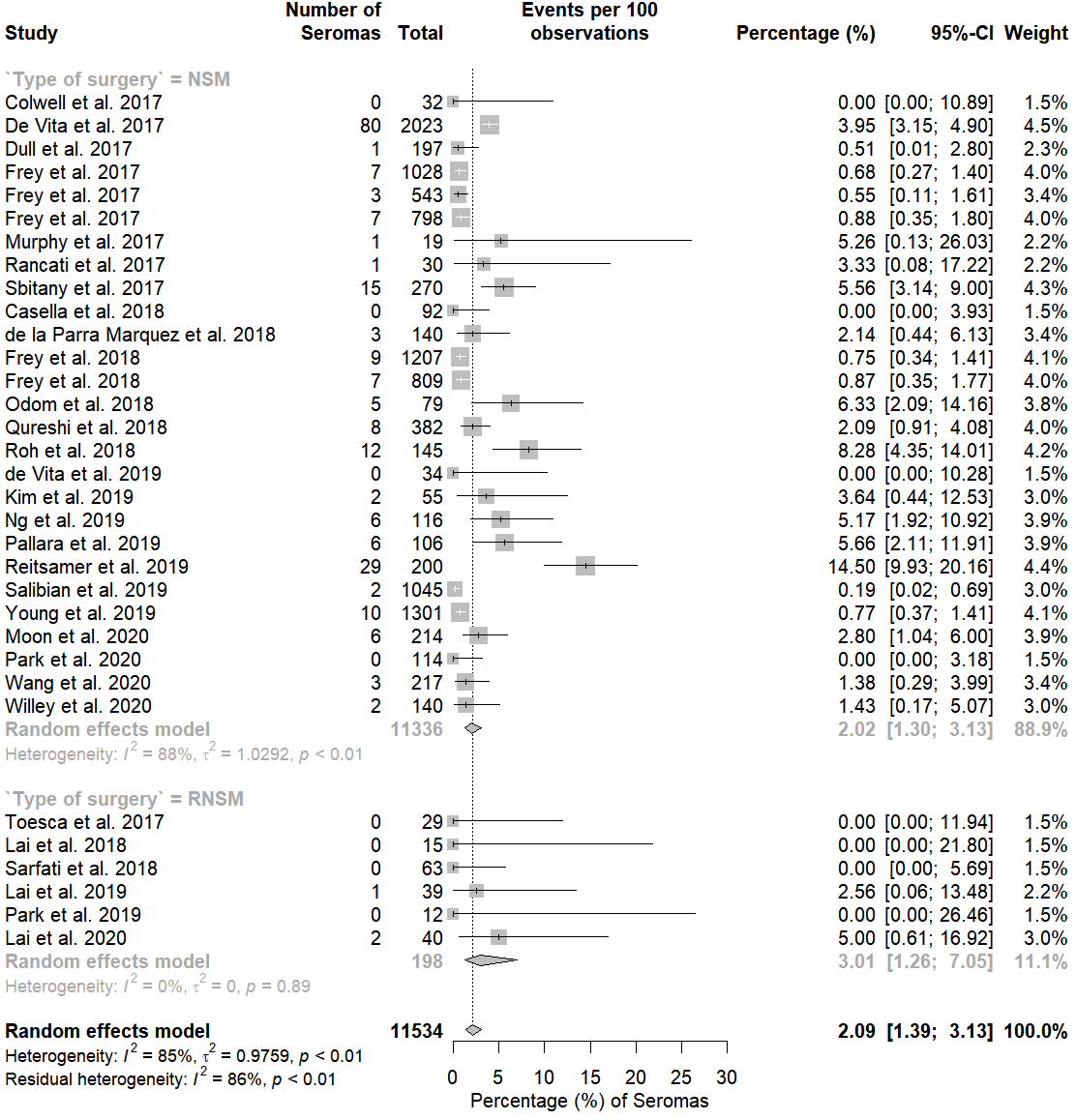 |
| --- |
| Supplementary Figure 5. Seroma. P = 0.421, (R)NSM = (Robotic) nipple sparing mastectomy, CI = confidence interval. |

# References

1. Frey JD, Choi M, Salibian AA, Karp NS. Comparison of Outcomes with Tissue Expander, Immediate Implant, and Autologous Breast Reconstruction in Greater Than 1000 Nipple-Sparing Mastectomies. Plast Reconstr Surg. 2017 Jun;139(6):1300–10.

2. Rancati AO, Angrigiani CH, Hammond DC, Nava MB, Gonzalez EG, Dorr JC, et al. Direct to Implant Reconstruction in Nipple Sparing Mastectomy. Plast Reconstr Surg - Glob Open. 2017 Jun;5(6):e1369.

3. Frey JD, Alperovich M, Levine JP, Choi M, Karp NS. Does Smoking History Confer a Higher Risk for Reconstructive Complications in Nipple-Sparing Mastectomy? Breast J. 2017 Jul;23(4):415–20.

4. Frey JD, Salibian AA, Choi M, Karp NS. Mastectomy Flap Thickness and Complications in Nipple-Sparing Mastectomy. Plast Reconstr Surg - Glob Open. 2017 Aug;5(8):e1439.

5. Mesdag V, Régis C, Tresch E, Chauvet M-P, Boulanger L, Collinet P, et al. Nipple sparing mastectomy for breast cancer is associated with high patient satisfaction and safe oncological outcomes. J Gynecol Obstet Hum Reprod. 2017 Oct;46(8):637–42.

6. Dull B, Conant L, Myckatyn T, Tenenbaum M, Cyr A, Margenthaler JA. Nipple-sparing mastectomies: Clinical outcomes from a single academic institution. Mol Clin Oncol. 2017 May;6(5):737–42.

7. Lago V, Maisto V, Gimenez-Climent J, Vila J, Vazquez C, Estevan R. Nipple-sparing mastectomy as treatment for patients with ductal carcinoma in situ: A 10-year follow-up study. Breast J. 2018 May;24(3):298–303.

8. Murphy BL, Boughey JC, Hieken TJ. Nipple-sparing Mastectomy for the Management of Recurrent Breast Cancer. Clin Breast Cancer. 2017 Jul;17(4):e209–13.

9. Chan YH-Y, Yau W-M, Cheung PS-Y. Oncological Safety and Technical Feasibility of Nipple-Sparing Mastectomy for Breast Cancer: The Hong Kong Experience. World J Surg. 2018 May;42(5):1375–83.

10. Colwell AS, Taylor E, Specht M, Orringer JS. Optimizing Nipple Position following Nipple-Sparing Mastectomy. Plast Reconstr Surg - Glob Open. 2017 Sep;5(9):e1490.

11. De Vita R, Zoccali G, Buccheri EM, Costantini M, Botti C, Pozzi M. Outcome Evaluation after 2023 Nipple-Sparing Mastectomies. Plast Reconstr Surg. 2017 Feb;139(2):335e-347e.

12. Sbitany H, Piper M, Lentz R. Prepectoral Breast Reconstruction. Plast Reconstr Surg. 2017 Sep;140(3):432–43.

13. Toesca A, Peradze N, Manconi A, Galimberti V, Intra M, Colleoni M, et al. Robotic nipple-sparing mastectomy for the treatment of breast cancer: Feasibility and safety study. Breast. 2017 Feb;31:51–6.

14. Venturi ML, Mesbahi AN, Copeland-Halperin LR, Suh VY, Yemc L. SPY Elite’s Ability to Predict Nipple Necrosis in Nipple-Sparing Mastectomy and Immediate Tissue Expander Reconstruction. Plast Reconstr Surg - Glob Open. 2017 May;5(5):e1334.

15. Frey JD, Choi M, Karp NS. The Effect of Neoadjuvant Chemotherapy Compared to Adjuvant Chemotherapy in Healing after Nipple-Sparing Mastectomy. Plast Reconstr Surg. 2017 Jan;139(1):10e-19e.

16. Hashem T, Farahat A. Thoracodorsal artery perforator flap as an autologous alternative to acellular dermal matrix. World J Surg Oncol. 2017 Dec;15(1):185.

17. Casella D, Di Taranto G, Marcasciano M, Sordi S, Kothari A, Kovacs T, et al. Nipple-sparing bilateral prophylactic mastectomy and immediate reconstruction with TiLoop(®) Bra mesh in BRCA1/2 mutation carriers: A prospective study of long-term and patient reported outcomes using the BREAST-Q. Breast [Internet]. 2018;39:8–13. Available from: https://pubmed.ncbi.nlm.nih.gov/29455110

18. Marquez MDLP, Fernandez-Riera R, Cardona HV, Flores JMR. Immediate implant replacement with DIEP flap: a single-stage salvage option in failed implant-based breast reconstruction. World J Surg Oncol [Internet]. 2018;16(1):80. Available from: https://pubmed.ncbi.nlm.nih.gov/29665804

19. Frey JD, Salibian AA, Levine JP, Karp NS, Choi M. Incision Choices in Nipple-Sparing Mastectomy: A Comparative Analysis of Outcomes and Evolution of a Clinical Algorithm. Plast Reconstr Surg [Internet]. 2018;142(6):826e-835e. Available from: https://pubmed.ncbi.nlm.nih.gov/30204677

20. Frey JD, Salibian AA, Karp NS, Choi M. The Impact of Mastectomy Weight on Reconstructive Trends and Outcomes in Nipple-Sparing Mastectomy: Progressively Greater Complications with Larger Breast Size. Plast Reconstr Surg [Internet]. 2018;141(6):795e-804e. Available from: https://pubmed.ncbi.nlm.nih.gov/29794693

21. Lai H-W, Chen S-T, Lin S-L, Chen C-J, Lin Y-L, Pai S-H, et al. Robotic Nipple-Sparing Mastectomy and Immediate Breast Reconstruction with Gel Implant: Technique, Preliminary Results and Patient-Reported Cosmetic Outcome. Ann Surg Oncol [Internet]. 2019;26(1):42–52. Available from: https://pubmed.ncbi.nlm.nih.gov/30109537

22. Odom EB, Parikh RP, Um G, Kantola SW, Cyr AE, Margenthaler JA, et al. Nipple-Sparing Mastectomy Incisions for Cancer Extirpation Prospective Cohort Trial: Perfusion, Complications, and Patient Outcomes. Plast Reconstr Surg [Internet]. 2018;142(1):13–26. Available from: https://pubmed.ncbi.nlm.nih.gov/29878989

23. Pek W-S, Tan B-K, Ru Ng YY, Kiak Mien Tan V, Rasheed MZ, Kiat Tee Tan B, et al. Immediate breast reconstruction following nipple-sparing mastectomy in an Asian population: Aesthetic outcomes and mitigating nipple-areolar complex necrosis. Arch Plast Surg [Internet]. 2018;45(3):229–38. Available from: https://pubmed.ncbi.nlm.nih.gov/29788682

24. Qureshi AA, Oliver JD, Parikh RP, Tenenbaum MM, Myckatyn TM. Salvage of Implant-Based Breast Reconstruction in Nipple-Sparing Mastectomies With Autologous Flaps. Aesthetic Surg J [Internet]. 2018;38(7):734–41. Available from: https://pubmed.ncbi.nlm.nih.gov/29360922

25. Roh TS, Kim JY, Jung BK, Jeong J, Ahn SG, Kim YS. Comparison of Outcomes between Direct-to-Implant Breast Reconstruction Following Nipple-Sparing Mastectomy through Inframammary Fold Incision versus Noninframammary Fold Incision. J Breast Cancer [Internet]. 2018;21(2):213–21. Available from: https://pubmed.ncbi.nlm.nih.gov/29963118

26. Sarfati B, Struk S, Leymarie N, Honart JF, Alkhashnam H, de Fremicourt KT, et al. Robotic Prophylactic Nipple-Sparing Mastectomy with Immediate Prosthetic Breast Reconstruction: A Prospective Study. Ann Surg Oncol. 2018 Sep;25(9):2579–86.

27. de Vita R, Buccheri EM, Villanucci A, Pozzi M. Breast Reconstruction Actualized in Nipple-sparing Mastectomy and Direct-to-implant, Prepectoral Polyurethane Positioning: Early Experience and Preliminary Results. Clin Breast Cancer [Internet]. 2019;19(2):e358–63. Available from: https://pubmed.ncbi.nlm.nih.gov/30691930

28. Houvenaeghel G, Bannier M, Rua S, Barrou J, Heinemann M, Troy A Van, et al. Breast cancer robotic nipple sparing mastectomy: evaluation of several surgical procedures and learning curve. World J Surg Oncol. 2019 Feb;17(1):27-019-1567-y.

29. Ito H, Ueno T, Suga H, Shiraishi T, Isaka H, Imi K, et al. Risk Factors for Skin Flap Necrosis in Breast Cancer Patients Treated with Mastectomy Followed by Immediate Breast Reconstruction. World J Surg [Internet]. 2019;43(3):846–52. Available from: https://pubmed.ncbi.nlm.nih.gov/30426185

30. Kim H, Park S-J, Woo K-J, Bang SI. Comparative Study of Nipple-Areola Complex Position and Patient Satisfaction After Unilateral Mastectomy and Immediate Expander-Implant Reconstruction Nipple-Sparing Mastectomy Versus Skin-Sparing Mastectomy. Aesthetic Plast Surg [Internet]. 2019;43(2):313–27. Available from: https://pubmed.ncbi.nlm.nih.gov/30746565

31. Lai H-W, Wang C-C, Lai Y-C, Chen C-J, Lin S-L, Chen S-T, et al. The learning curve of robotic nipple sparing mastectomy for breast cancer: An analysis of consecutive 39 procedures with cumulative sum plot. Eur J Surg Oncol [Internet]. 2019;45(2):125–33. Available from: https://pubmed.ncbi.nlm.nih.gov/30360987

32. Ng T, Knowles S, Brackstone M, Doherty C. Mastectomy flap necrosis after nipple-sparing mastectomy and immediate implant-based reconstruction: An evaluation of tumescence and sharp dissection technique on surgical outcomes. Breast J [Internet]. 2019;25(6):1079–83. Available from: https://pubmed.ncbi.nlm.nih.gov/31359567

33. Pallara T, Cagli B, Fortunato L, Altomare V, Loreti A, Grasso A, et al. Direct-To-Implant and 2-Stage Breast Reconstruction After Nipple Sparing Mastectomy: Results of a Retrospective Comparison. Ann Plast Surg [Internet]. 2019;10.1097/SAP.0000000000001893. Available from: https://pubmed.ncbi.nlm.nih.gov/31246667

34. Park HS, Lee J, Lee DW, Song SY, Lew DH, Kim S Il, et al. Robot-assisted Nipple-sparing Mastectomy with Immediate Breast Reconstruction: An Initial Experience. Sci Rep [Internet]. 2019;9(1):15669. Available from: https://pubmed.ncbi.nlm.nih.gov/31666551

35. E.M. Q, M. BB, M. K, S. V, R. M, R. Z, et al. Successful Immediate Staged Breast Reconstruction with Intermediary Autologous Lipotransfer in Irradiated Patients. Breast Care (Basel) [Internet]. 2019;145(2):S1072-7515(20)30116-2. Available from: https://pubmed.ncbi.nlm.nih.gov/31491673

36. Reitsamer R, Peintinger F, Klaassen-Federspiel F, Sir A. Prepectoral direct-to-implant breast reconstruction with complete ADM or synthetic mesh coverage - 36-Months follow-up in 200 reconstructed breasts. Breast [Internet]. 2019;48:32–7. Available from: https://pubmed.ncbi.nlm.nih.gov/31491673

37. Salibian AA, Frey JD, Bekisz JM, Karp NS, Choi M. Ischemic Complications after Nipple-sparing Mastectomy: Predictors of Reconstructive Failure in Implant-based Reconstruction and Implications for Decision-making. Plast Reconstr surgery Glob open [Internet]. 2019;7(5):e2280–e2280. Available from: https://pubmed.ncbi.nlm.nih.gov/31333984

38. Tasoulis M-K, Agusti A, Karakatsanis A, Montgomery C, Marshall C, Gui G. The Use of Hydrodissection in Nipple- and Skin-sparing Mastectomy. Plast Reconstr Surg - Glob Open. 2019 Nov;7(11):e2495.

39. Yang C-Q, Ji F, Gao H-F, Zhang L-L, Yang M, Zhu T, et al. The Role of Sharp Dissection in Nipple-Sparing Mastectomy: A Safe Procedure with No Necrosis of the Nipple-Areolar Complex. Cancer Manag Res [Internet]. 2019;11:10223–8. Available from: https://pubmed.ncbi.nlm.nih.gov/31824192

40. Young WA, Degnim AC, Hoskin TL, Jakub JW, Nguyen M-D, Tran N V, et al. Outcomes of > 1300 Nipple-Sparing Mastectomies with Immediate Reconstruction: The Impact of Expanding Indications on Complications. Ann Surg Oncol [Internet]. 2019;26(10):3115–23. Available from: https://pubmed.ncbi.nlm.nih.gov/31342370

41. Chan YH-Y, Yue IK-H, Ho C-M, Cheung PS-Y. The Use of Serratus Anterior Fascial Flap in Integrated Mastectomy and Implant Reconstruction. World J Surg [Internet]. 2020;44(3):825–30. Available from: https://pubmed.ncbi.nlm.nih.gov/31732761

42. Lai H-W, Chen S-T, Tai C-M, Lin S-L, Lin Y-J, Huang R-H, et al. Robotic- Versus Endoscopic-Assisted Nipple-Sparing Mastectomy with Immediate Prosthesis Breast Reconstruction in the Management of Breast Cancer: A Case-Control Comparison Study with Analysis of Clinical Outcomes, Learning Curve, Patient-Reported Aestheti. Ann Surg Oncol [Internet]. 2020;10.1245/s10434-020-08223–0. Available from: https://pubmed.ncbi.nlm.nih.gov/32016631

43. Moon K-C, Baek S-O, Yoon E-S, Lee B-I, Park S-H. Predictors affecting complications and aesthetic outcomes in autologous breast reconstruction with free muscle-sparing transverse rectus abdominis myocutaneous flaps. Microsurgery [Internet]. 2020;40(1):38–43. Available from: https://pubmed.ncbi.nlm.nih.gov/30793787

44. Park KU, Weiss A, Rosso K, Yi M, Hunt K, Kuerer H, et al. Use of Mammographic Measurements to Predict Complications After Nipple-Sparing Mastectomy in BRCA Mutation Carriers. Ann Surg Oncol [Internet]. 2020;27(2):367–72. Available from: https://pubmed.ncbi.nlm.nih.gov/31399819

45. Salibian AA, Bekisz JM, Frey JD, Miller B, Choi M, Karp NS. Prophylactic nipple‐sparing mastectomy in young previvors: Examining decision‐making, reconstructive outcomes, and patient satisfaction in BRCA+ patients under 30. Breast J. 2020 May;26(5):971–5.

46. Seki H, Sakurai T, Maeda Y, Oki N, Aoyama M, Yamaguchi R, et al. Utility of the periareolar incision technique for breast reconstructive surgery in patients with breast cancer. Surg Today [Internet]. 2020;10.1007/s00595-020-01975-y. Available from: https://pubmed.ncbi.nlm.nih.gov/32052184

47. Valero MG, Muhsen S, Moo T-A, Zabor EC, Stempel M, Pusic A, et al. Increase in Utilization of Nipple-Sparing Mastectomy for Breast Cancer: Indications, Complications, and Oncologic Outcomes. Ann Surg Oncol [Internet]. 2020;27(2):344–51. Available from: https://pubmed.ncbi.nlm.nih.gov/31823173

48. Wang M, Huang J, Chagpar AB. Is nipple sparing mastectomy associated with increased complications, readmission and length of stay compared to skin sparing mastectomy? Am J Surg. 2020 Jun;219(6):1030–5.

49. Willey SC, Fan KL, Luvisa K, Graziano FD, Lau SHY, Black CK, et al. Predicting Ischemic Complications in the Inframammary Approach to Nipple-Sparing Mastectomy: The Midclavicular-to-Inframammary Fold Measurement. Plast Reconstr Surg [Internet]. 2020;145(2):251e-262e. Available from: https://pubmed.ncbi.nlm.nih.gov/31985611
